# Clinical and Computational Analysis of Left Subclavian Artery Coverage on High-Risk Blunt Thoracic Aortic Injury

**DOI:** 10.3390/jcm15166269

**Published:** 2026-08-13

**Authors:** Alireza Jabbarinick, Mohammadebrahim Varan, Hamidreza Pouraliakbar, Nima Rahmati, Rezvan Dadras, Jamal Moosavi, Bahram Mohebbi, Sepehr Jamalkhani, Somayyeh Barati, Mona Alimohammadi, Parham Sadeghipour

**Affiliations:** 1 Department of Mechanical Engineering, K. N. Toosi University of Technology, Tehran 19991-43344, Iran; alirezajabbarinick7158@gmail.com (A.J.); n_rahmati@yahoo.com (N.R.);; 2 Department of Mechanical Engineering, College of Engineering, University of Tehran, Tehran 14174-66191, Iran; 3 Vascular Disease and Thrombosis Research Center, Rajaie Cardiovascular Institute, Tehran 19956-14331, Iran

**Keywords:** computational fluid dynamics, blunt thoracic aortic injury, thoracic endovascular aortic repair, windkessel model, hemodynamics, swirling strength

## Abstract

**Background/Objectives:** Blunt thoracic aortic injury (BTAI) is a rare, highly lethal trauma typically occurring at the aortic isthmus. Advanced BTAI is primarily treated with thoracic endovascular aortic repair (TEVAR). Because emergent surgical debranching is rarely feasible, management depends heavily on patient anatomy, especially regarding the left subclavian artery (LSA). Patient-specific computational fluid dynamics (CFD) models offer critical insights into periprocedural planning and outcome prediction. **Methods:** This study investigates hemodynamic changes in a patient-specific BTAI case following intentional LSA coverage by a stent graft. Three-dimensional patient-specific models were coupled with RCR-Windkessel boundary conditions for both pre- and post-procedural imaging data to simulate blood flow in each scenario. **Results:** Post-intervention, flow distribution improved significantly; relative perfusion to the brachiocephalic trunk and left common carotid artery increased by 3.51% and 4.02%, respectively, alongside an elevated overall pressure throughout the entire computational domain. However, regions with high oscillatory, low magnitude shear (HOLMES), specifically wall areas with values < 0.3 Pa, expanded post-stenting. This warrants careful monitoring during follow-ups, given the associated risk of thrombus formation. Furthermore, time-averaged swirling strength (TASS) variation along the aorta decreased (standard deviation dropped from 1.8610 to 1.3835), indicating stabilized flow within the stented region, while normalized swirling strength increased distally. **Conclusions:** This study establishes an effective, non-invasive framework for assessing pre- and post-TEVAR hemodynamics. It demonstrates that LSA coverage induces uniformly elevated pressure and alters wall shear stress and helicity indices, highlighting the need for future research into pharmacological management to optimize long-term outcomes.

## 1. Introduction

Blunt thoracic aortic injury (BTAI) is the second most common cause of fatal thoracic trauma [1]. Approximately 80–85% of BTAI victims die before hospital admission, while 40% of survivors die during hospitalization [1,2,3]. Although BTAI is a fatal condition, it remains relatively uncommon, accounting for about 1.5% of all thoracic traumas [4]. BTAI typically results from a combination of stretching and shearing forces caused by rapid deceleration and compression of the aorta between the spine and anterior chest wall [4,5]. This mechanism is frequently observed in road traffic accidents, often due to sudden impacts from seat belts or steering wheels, leading to hemorrhage at the injury site [3].

BTAI is classified based on the extent of aortic wall damage: Grade I (intimal tear), Grade II (intramural hematoma), Grade III (pseudoaneurysm), and Grade IV (rupture) [6]. Among these, pseudoaneurysm (Grade III) is the most common [7], where wall disruption leads to pseudoaneurysm formation and local coarctation at the injury site [8]. Such narrowing causes aortic obstruction and alters blood flow within the descending aorta [8]. The resulting high-velocity swirling flows impinge on the aortic wall, inducing discontinuous wall shear stress (WSS) and increasing the risk of rupture [9,10,11].

Early diagnosis and proper management are crucial for improving BTAI outcomes [3]. Thoracic endovascular aortic repair (TEVAR) has emerged as a rapid and preferred endovascular treatment, offering fewer perioperative complications and favorable long-term mortality rates [12,13]. Despite these advantages, thoracic stent grafts were originally designed for aneurysmal aortic conditions and are often unsuitable for the narrower diameters and sharp angulations of the aortic arch [14]. Additional TEVAR-related complications include endoleak, distal stent thrombosis, and occlusion [12]. Therefore, beyond clinical and follow-up assessments, comparative analyses of pre- and post-stenting conditions are essential to optimize treatment strategies and long-term outcomes.

In recent years, only a few numerical studies have examined BTAI mechanisms and rupture locations. For example, Wei et al. [15] developed a detailed finite element (FE) model simulating road accidents that included the heart, aorta, and surrounding organs, predicting rupture at the aortic isthmus. Similarly, Nan et al. [16] created a patient-specific FE model to assess aortic behavior during vehicle collisions and found that the maximum stress occurred at the aortic isthmus. Their study concluded that arch bending, rather than ascending aortic stretching, is the dominant factor in BTAI. Other studies have also confirmed that the aortic isthmus has the highest risk of rupture and that bending motion within the arch is the most critical mechanism leading to injury [17,18]. Given this, the most common injury site often requires a stent landing zone that covers the left subclavian artery (LSA) in typical BTAI cases [19].

However, intentional coverage of the LSA introduces notable clinical challenges. Evidence has demonstrated that sacrificing the LSA origin potentially compromises blood supply to critical vascular territories, risking left upper extremity ischemia, posterior cerebral stroke, and spinal cord ischemia [20]. To mitigate these risks, a routine revascularization strategy serves as the standard procedure protocol for some surgeons, who routinely restore blood flow to the LSA to minimize the risk of neurological complications. Conversely, the selective coverage strategy allows the artery to be covered by endovascular grafting without primary revascularization, provided the patient’s overall cerebrovascular anatomy, including vertebral artery dominance and the completeness of the circle of Willis, is thoroughly evaluated to ensure adequate collateral pathway [21]. This clinical rationale is strongly supported by the Society for Vascular Surgery Clinical Practice Guidelines, which endorse prioritizing immediate endovascular aortic stabilization via a selective coverage strategy over routine, time-consuming revascularization procedures for BTAI patients during the acute trauma phase [22].

Computational fluid dynamics (CFD) provides a non-invasive approach for quantifying hemodynamic parameters that cannot be directly measured in vivo. It offers valuable insights for pre- and post-intervention analysis and supports the prediction of post-treatment hemodynamic responses, aiding clinical decision-making [23,24,25,26,27,28]. However, despite the strong potential of CFD in vascular research, to the authors’ knowledge, no prior study has conducted a fully patient-specific CFD simulation analyzing both pre- and post-intervention conditions for a clinical BTAI case involving LSA coverage.

In this context, the present work aims to bridge this gap by performing a detailed CFD-based investigation of a patient-specific BTAI case treated with TEVAR where the LSA was intentionally covered by a stent graft. This study provides novel quantitative insights into hemodynamic alterations before and after intervention, highlighting potential implications for clinical planning and long-term management of BTAI patients.

## 2. Materials and Methods

### 2.1. Clinical Data

Medical imaging data were obtained from a 59-year-old male patient who sustained injuries in a road traffic accident. The case was investigated at Rajaie Cardiovascular Institute under ethical approval number IR.IUMS.FMD.REC.1401.257. Following the diagnosis of BTAI, TEVAR was performed according to the latest clinical recommendations [29]. A 34  ×  161 mm Zenith Thoracic Endovascular Graft (Cook Medical, Bloomington, IN, USA) was deployed, intentionally covering the LSA. Vertebral artery anatomy, including left vertebral artery dominance, was routinely assessed during preprocedural planning. The LSA was subsequently coiled with two MReye Embolization Coils (8 mm and 10 mm, Cook Medical, Bloomington, IN, USA) to minimize the risk of type II endoleak.

The procedure was technically successful, and postoperative imaging confirmed complete exclusion of the aortic pathology. Follow-up imaging at 1, 6, and 12 months demonstrated no residual defects, endoleaks, or procedure-related complications. The patient remained asymptomatic and exhibited no signs of upper limb ischemia throughout the follow-up period.

### 2.2. Segmentation and Meshing

The computational domain extended from the beginning of the aortic arch to the proximal renal arteries for both pre- and post-intervention models. Contrast-enhanced computed tomography (CT) images were available for both cases, comprising 1953 and 1731 Digital Imaging and Communications in Medicine (DICOM) slices, respectively, with a slice interval of 0.4 mm. The images were imported into MIMICS Research 21.0 (Materialise, Leuven, Belgium) for three-dimensional reconstruction.

**Figure 1:**
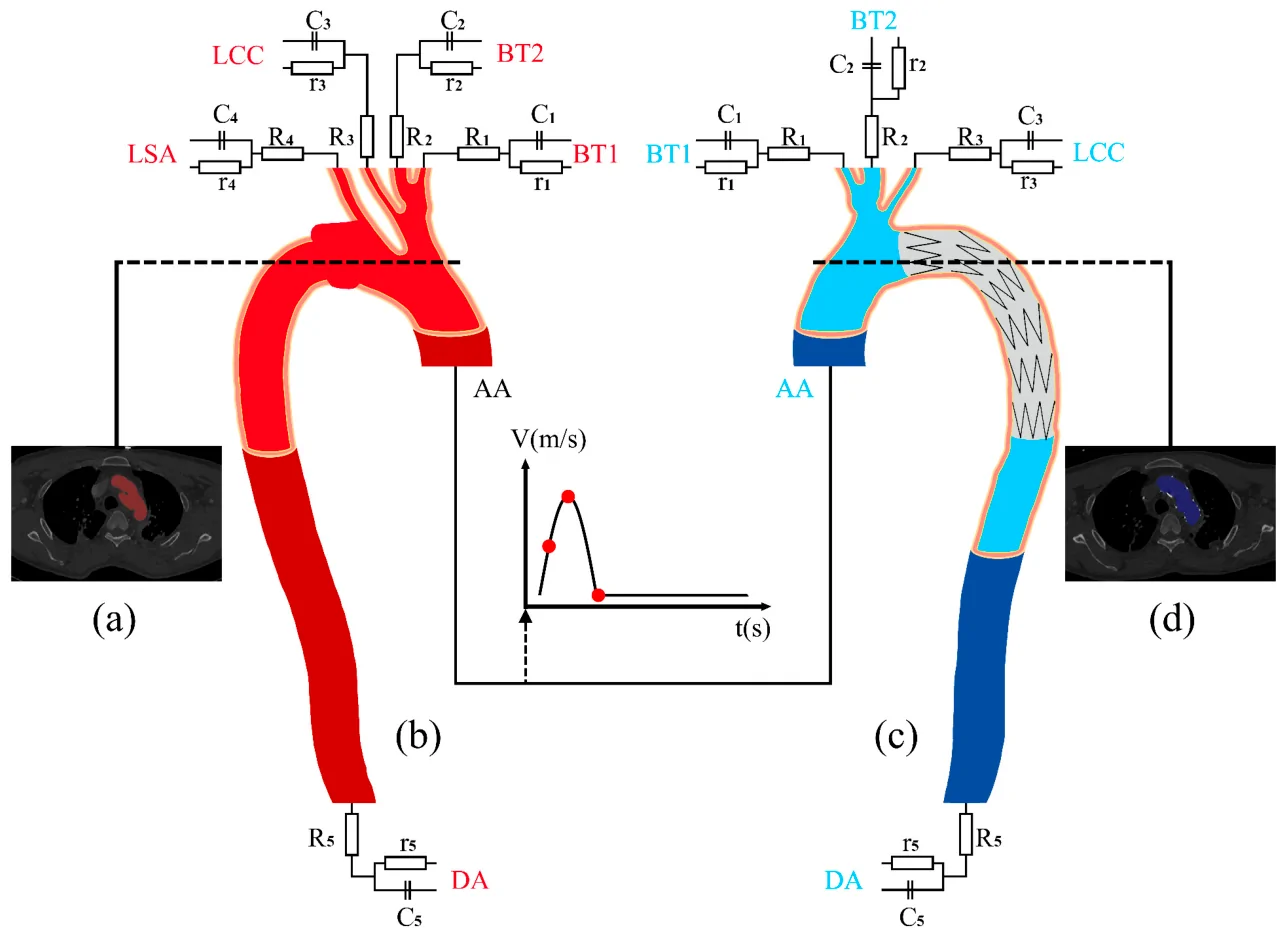
Schematics of geometries and boundary conditions. (**a**,**d**): computed tomography images of before and after stenting instances. (**b**,**c**): fluid domains and RCR boundary conditions for pre- and post-instances.

Segmentation included the aortic arch, supra-aortic branches, and major downstream vessels. To ensure accurate volumetric representation of the three-dimensional (3D) geometry, smoothing and wrapping operations were applied. Using CATIA V21 (Dassault Systems, Vélizy-Villacoublay, France), cross-sectional planes perpendicular to the z-axis were created at the beginning of the arch, at the renal proximal section, and along each supra-aortic branch, as shown in Figure 1. Cross-sectional planes of corresponding branches were defined in an identical manner across both pre- and post-intervention geometries, and boundaries were labeled accordingly (Figure 1b,c). The reconstructed 3D geometries were discretized using ANSYS Meshing 2019 R3 (ANSYS Inc., Canonsburg, PA, USA). The computational grid consisted of tetrahedral elements with five prism layers applied along the vessel walls to capture near-wall flow gradients. Mesh independence analysis was performed using three mesh densities, and the medium grid, containing approximately 400,000 elements, was selected. Increasing the mesh density to 800,000 elements resulted in less than a 1% variation in peak velocity and Time-averaged wall shear stress (TAWSS), confirming mesh independence. To ensure numerical accuracy and solver stability, mesh quality indices were carefully monitored, yielding a maximum cell skewness of 0.77, a minimum orthogonal quality of 0.24, and an average aspect ratio of 2.3, all of which fall well within the highly acceptable ranges for complex aortic flow simulations.

### 2.3. Boundary Conditions

Due to the emergency nature of BTAI and the time-sensitive aspects of diagnosis and treatment, patient-specific inlet velocity data were not available. To maintain consistency between the pre- and post-intervention models, an idealized half-sine waveform with a cardiac cycle duration of 0.731 s, derived from a previous TEVAR study [30], was applied as the inlet boundary condition for both cases. For the outlet boundaries, three-element Windkessel (RCR) models were employed. The parameters were initially tuned based on the pre-intervention case following the methodology proposed by Alimohammadi et al. [23], and the final values are summarized in Table 1.

To ensure consistency and minimize computational cost, these tuned parameters were also applied to the post-intervention simulations. In accordance with previous research findings [30], which reported negligible differences between deformable and rigid wall models in similar simulations, the aortic wall was assumed to be rigid with a no-slip boundary condition for all cases. This simplification allowed for reduced computational demand while maintaining acceptable physiological accuracy.

### 2.4. Simulation

The governing Cauchy momentum and continuity equations were solved using ANSYS CFX 2019 R3 (ANSYS Inc., Canonsburg, PA, USA) under the aforementioned boundary conditions. To prevent numerical instability and to effectively capture secondary flow phenomena within the aorta, the Shear Stress Transport (SST) turbulence model with a turbulence intensity of 1% was implemented for both scenarios. The SST model was specifically chosen due to its robust performance in capturing transitional flow, flow separation, and adverse pressure gradients induced by the sudden geometric expansions and contractions characteristic of pseudoaneurysms and stent grafts. Also, the localized Reynolds number was evaluated at the narrowest section of the aorta, reaching a peak systolic value of approximately 4100 and confirming SST model implementation. Blood was modeled as an incompressible, non-Newtonian fluid using the Carreau–Yasuda model [31], with a density of 1056 kg/m^3^. The viscosity μ was defined according to Equation (1):

(1) $$\mu =\left(\mu_{0}-\mu_{\infty }\right){\left(1+{\left(\lambda_{CY}\gamma^{\prime }\right)}^{a}\right)}^{(m-1)/a}+\mu_{\infty }$$

wherein $\mu_{0}$, $\mu_{\infty }$, $\lambda_{CY}$, $\gamma^{\prime }$, a and m were named as low shear viscosity, high shear viscosity, time constant, shear rate, Yasuda exponent, and power law index, respectively; their values are reported in Table 2 [31].

The fluid domain was initialized with diastolic pressure conditions, and simulations were carried out for three consecutive cardiac cycles to ensure a periodic steady state. A uniform time-step size of 0.002 s was applied, and the convergence criterion for the root-mean-square residual was set to 1 × 10^−5^ for all governing equations.

### 2.5. Flow Analysis

#### 2.5.1. Velocity and Pressure Metrics

In this study, streamlines with identical settings and ranges were extracted and displayed for both pre- and post-intervention cases. Additionally, two cross-sectional planes were selected in the pre-intervention geometry: one located at the midpoint of the pseudoaneurysm and the other immediately distal to it. Corresponding cross-sections at the exact same locations were sampled in the post-intervention case to enable direct comparison. Relative flow distribution at each boundary was calculated using Equation (2).

(2) $$Relative\;flow\;distribution\;at\;a\;boundary\;=\frac{\;flow\;distribution\;for\;one\;cardiac\;cycle\;at\;a\;boundary}{flow\;distribution\;for\;one\;cardiac\;cycle\;at\;AA}$$

The same visualization parameters and fixed value ranges were applied to display pressure fields for both cases. It is important to note that pressure and velocity data were averaged over three critical cardiac phases. The fixed ranges used for these comparisons were chosen solely for better visual contrast and do not represent the absolute maximum or minimum values.

#### 2.5.2. Wall Shear Stress Indices

Wall shear stress (WSS) values and derived hemodynamic indices play a significant role in understanding patient-specific vascular conditions [23]. Time-averaged wall shear stress (TAWSS), oscillatory shear index (OSI), and high oscillatory low magnitude shear (HOLMES) were computed over all time steps within the cardiac cycle, after reaching periodic steady state, using Equations (3), (4), and (5), respectively:

(3) $$\mathrm{TAWSS}\;=\frac{1}{T}\int_{t}^{\;\;\;t+T}\;\left|\tau \right|dt,$$

(4) $$OSI=\frac{1}{2}\left(1-\frac{\left|\left(\frac{1}{T}\right)\int_{t}^{t+T}\;\tau dt\right|}{\left(\frac{1}{T}\right)\int_{t}^{t+T}\;\left|\tau \right|dt}\right),$$

(5) HOLMES = TAWSS ×(0.5−OSI)

In which $\left|\tau \right|$ indicates the WSS vector at each timestep or instantaneous WSS vector at each node. t and T=0.731s represent the start time of the cardiac cycle and cardiac cycle durations, respectively [25]. Notably, for a more accurate investigation of lower HOLMES values, the percentage of the area of the aortic wall with a lower value than a specific amount (between 0 and 1 Pa) was calculated and visualized. Equation (6) demonstrates the formula to calculate the percentage of area of the aortic wall with lower values than constant:

(6) $$Percentage\;of\;area\;of\;the\;aortic\;wall\;with\;lower\;value\;than\;constant=\frac{area\;of\;the\;aortic\;wall\;with\;lower\;value\;than\;constant}{area\;of\;the\;aortic\;wall}$$

#### 2.5.3. Helicity Metrics

Swirling Strength (SS) is a hemodynamic parameter initially introduced by Adrian et al. [32] to quantify the intensity of rotational blood flow as it moves through various regions toward downstream vessels. The validity of SS as a measure of circulation intensity was subsequently confirmed by Qi-Qiang et al. [33], who demonstrated that higher absolute SS values correspond to stronger swirling flow. Mathematically, SS is defined as the imaginary part of the complex eigenvalue of the velocity gradient tensor, as shown in Equation (7), where the velocity components in the x, y, and z directions are denoted by U, V, and W, respectively.

(7) $$\vec{J}=\nabla \vec{U}=\left[\begin{array}{c} \partial U/\partial x\;\;\;\;\partial U/\partial y\;\;\;\;\partial U/\partial z\\ \partial V/\partial x\;\;\;\;\partial V/\partial y\;\;\;\;\partial V/\partial z\\ \partial W/\partial x\;\;\;\;\partial W/\partial y\;\;\;\;\partial W/\partial z \end{array}\right]$$

For both pre- and post-intervention cases, thirty-eight horizontal cross-sectional planes were created along the aorta with a uniform vertical spacing of 6.25 mm, ensuring that each plane in one case corresponded to a plane in the other case. The inclusion of these additional planes enhances the accuracy of the analysis and reduces discretization errors. It is important to note that these thirty-eight planes are distinct from the two cross-sections used in the velocity and pressure analyses. Plane number 14 was identified as the first plane distal to the stent in the post-TEVAR case. The Time-Averaged Swirling Strength (TASS) was calculated on each of these planes to represent the average SS over the cardiac cycle, while the Volume-Averaged Swirling Strength (VASS) was computed across the entire computational domain. Normalized versions of these metrics, such as Normalized TASS (NTASS) and Normalized VASS (NVASS), were obtained by dividing TASS and VASS values by their respective maximum values within each case.

## 3. Results

### 3.1. Velocity and Streamline Distribution

**Figure 2:**
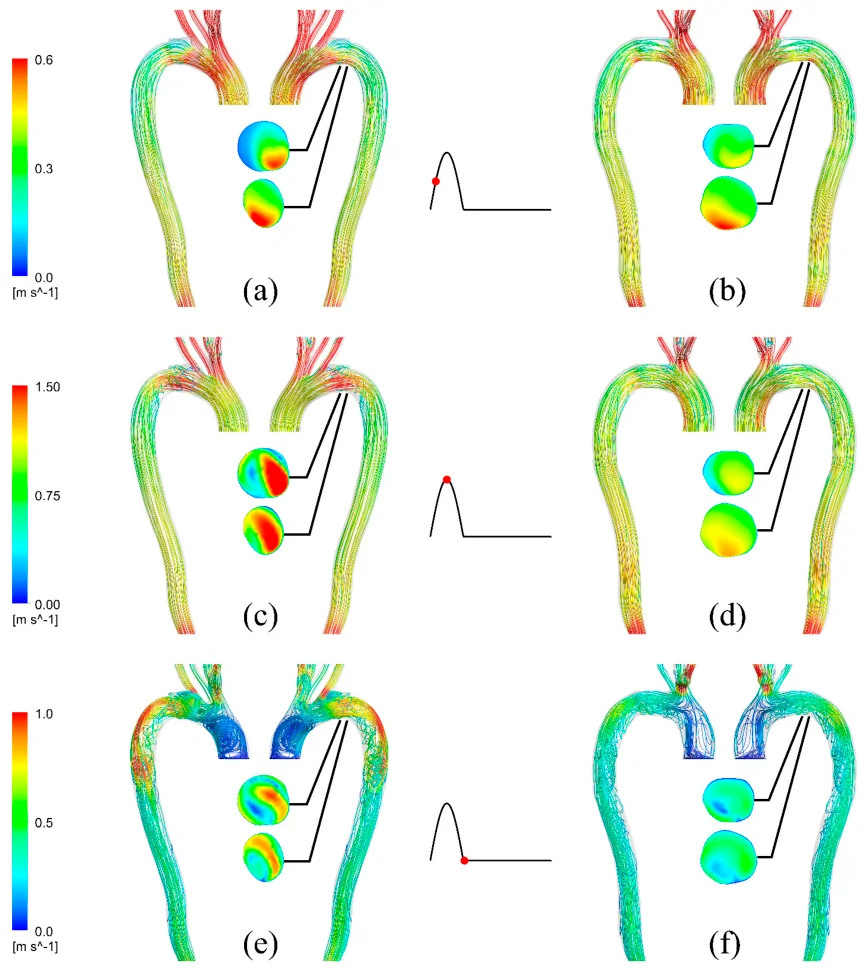
Streamline distribution and cross-sectional velocity on anterior and posterior views for before and after intervention instances. (**a**,**b**) Mid-systole, (**c**,**d**) peak-systole, and (**e**,**f**) diastole.

Forward and backward streamline patterns at three critical cardiac time points are shown in Figure 2. In both pre- and post-stenting cases during mid-systole (Figure 2a,b), the highest velocities are observed within the arterial branches, while the lowest velocities occur at the site of the pseudoaneurysm. Additionally, the flow within the aortic arch up to the distal end of the pseudoaneurysm appears non-uniform. Figure 2c,d illustrates velocity magnitudes during peak systole at the same anatomical locations as mid-systole, confirming similar regions of maximum and minimum velocity. During diastole (Figure 2e,f), the maximum velocity shifts to the distal end of the aortic arch. As blood enters the pseudoaneurysm, it experiences an initial reduction in velocity accompanied by disturbed, non-laminar flow. Upon exiting the pseudoaneurysm, velocity increases again, particularly during mid-systole and diastole.

At all three examined time points, the post-intervention case shows a general reduction in velocity magnitude throughout the arch as well as improved flow uniformity. This improvement in flow disturbances is most pronounced during peak systole. Two cross-sectional planes are highlighted for both cases in Figure 2: one at the pseudoaneurysm site and another immediately distal to it. These sections demonstrate flow homogenization and velocity reduction after stenting. Moreover, during mid- and peak systole, velocity increases in the descending aorta (DA), an effect that is more marked at peak systole due to higher inlet velocities.

A notable increase in velocity is also observed at the brachiocephalic trunk (BT) during diastole. Table 3 presents relative perfusion values averaged over a cardiac cycle for each case. Following intervention, relative perfusion increases in all branches except the LSA, which is occluded by the stent graft. Perfusion changes are particularly prominent in the branches of the aortic arch compared to the descending aorta (DA), with increases of 3.51%, 4.02%, and 2.15% observed in the BT, left common carotid (LCC), and DA, respectively.

### 3.2. Pressure Distribution

**Figure 3:**
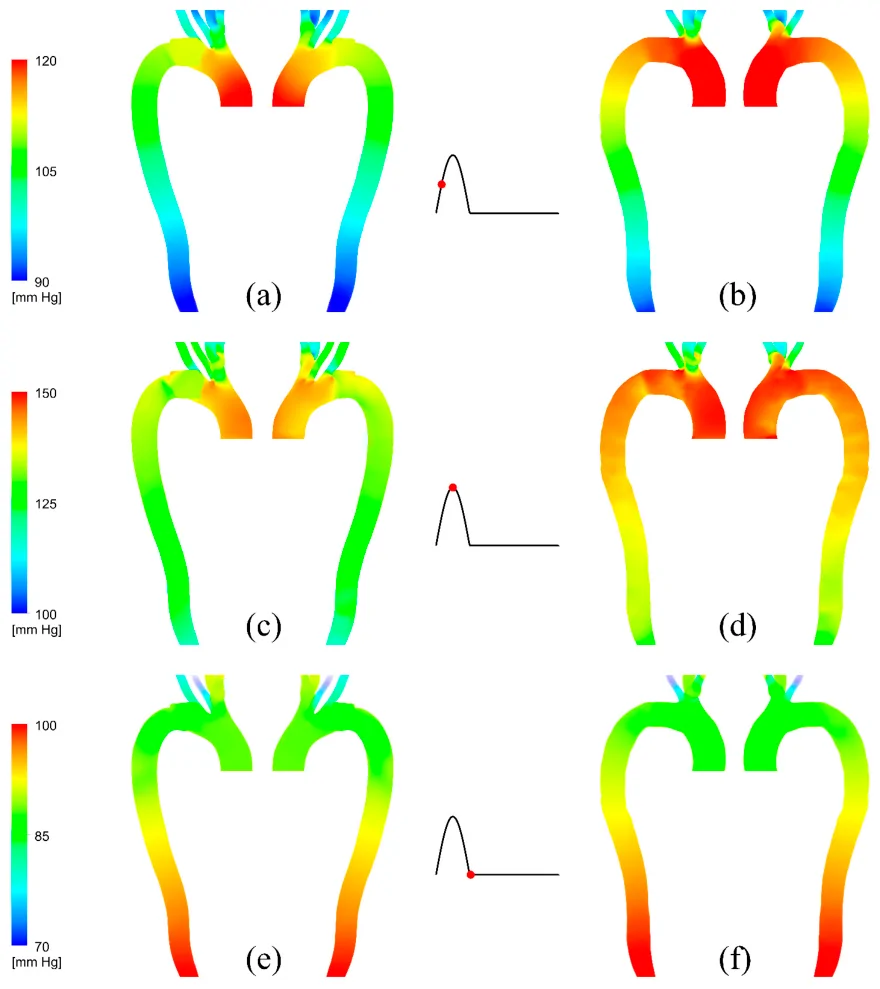
Pressure distribution on anterior and posterior views for before and after intervention instances. (**a**,**b**) Mid-systole, (**c**,**d**) peak-systole, and (**e**,**f**) diastole.

Pressure distributions in the analyzed section of the aorta for the pre- and post-intervention cases at the three previously mentioned cardiac phases are shown in Figure 3. During mid-systole (Figure 3a,b) and peak systole (Figure 3c,d), the maximum and minimum visible pressure values for both cases occur at the proximal part of the aortic arch and within the branches, respectively. In diastole (Figure 3e,f), within the chosen range, the highest-pressure magnitude is observed in the DA, while the lowest appears in the LCC. At all three time points, pressure increases throughout the aorta post-intervention compared to the pre-intervention case. Additionally, the pressure gradient along the aorta, from the semilunar valve to the DA, increased following stenting. Furthermore, the abrupt pressure drop observed at the pseudoaneurysm site and its distal region before intervention was eliminated after stenting, resulting in a more uniform pressure distribution along the aorta. Also, it should be noted that the pressure gradient along the pseudoaneurysm reduced from 6.8 mmHg to 1.6 mmHg after TEVAR.

### 3.3. Wall Shear Stress Indices

**Figure 4:**
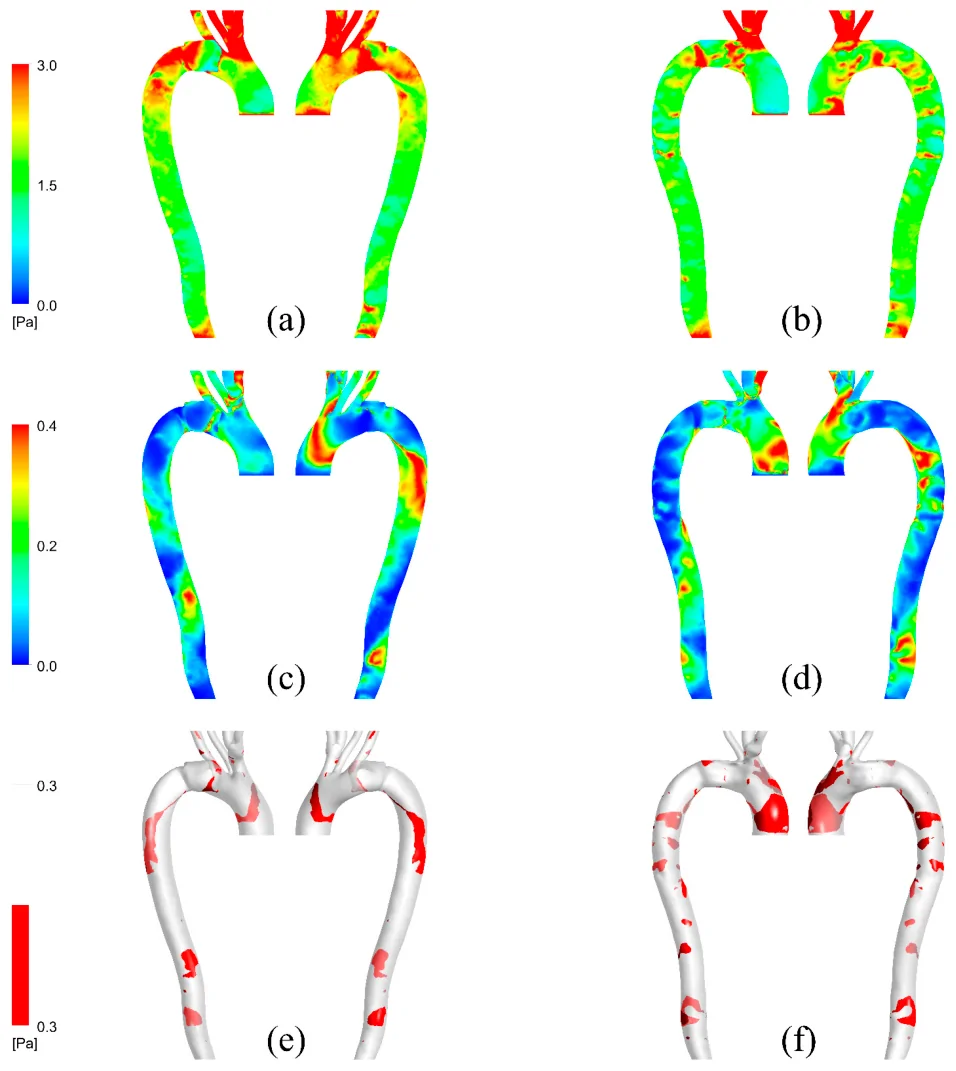
Wall shear stress indices on anterior and posterior views for before and after intervention instances. (**a**,**b**) TAWSS, (**c**,**d**) OSI, and (**e**,**f**) HOLMES.

The highest TAWSS values on the vessel wall (Figure 4a,b) are observed in both cases within the branches, DA, and adjacent to the semilunar valve. In the post-intervention case, TAWSS is diffusely reduced at the beginning of the pseudoaneurysm, the mid-portion, and the distal aortic arch. Notably, in the posterior view of the pre-intervention case, regions of low TAWSS at the proximal arch have expanded, whereas low TAWSS within the pseudoaneurysm disappeared after stenting. Before stenting (Figure 4c,d), zones of high OSI appear at the proximal and distal aortic arch and DA in the anterior view. Post-intervention, OSI decreases in these arch regions but increases in the DA. In the posterior view, OSI increases near the proximal arch after intervention but decreases toward the distal arch. High and concentrated OSI regions emerge scattered along the DA after stenting.

**Figure 5:**
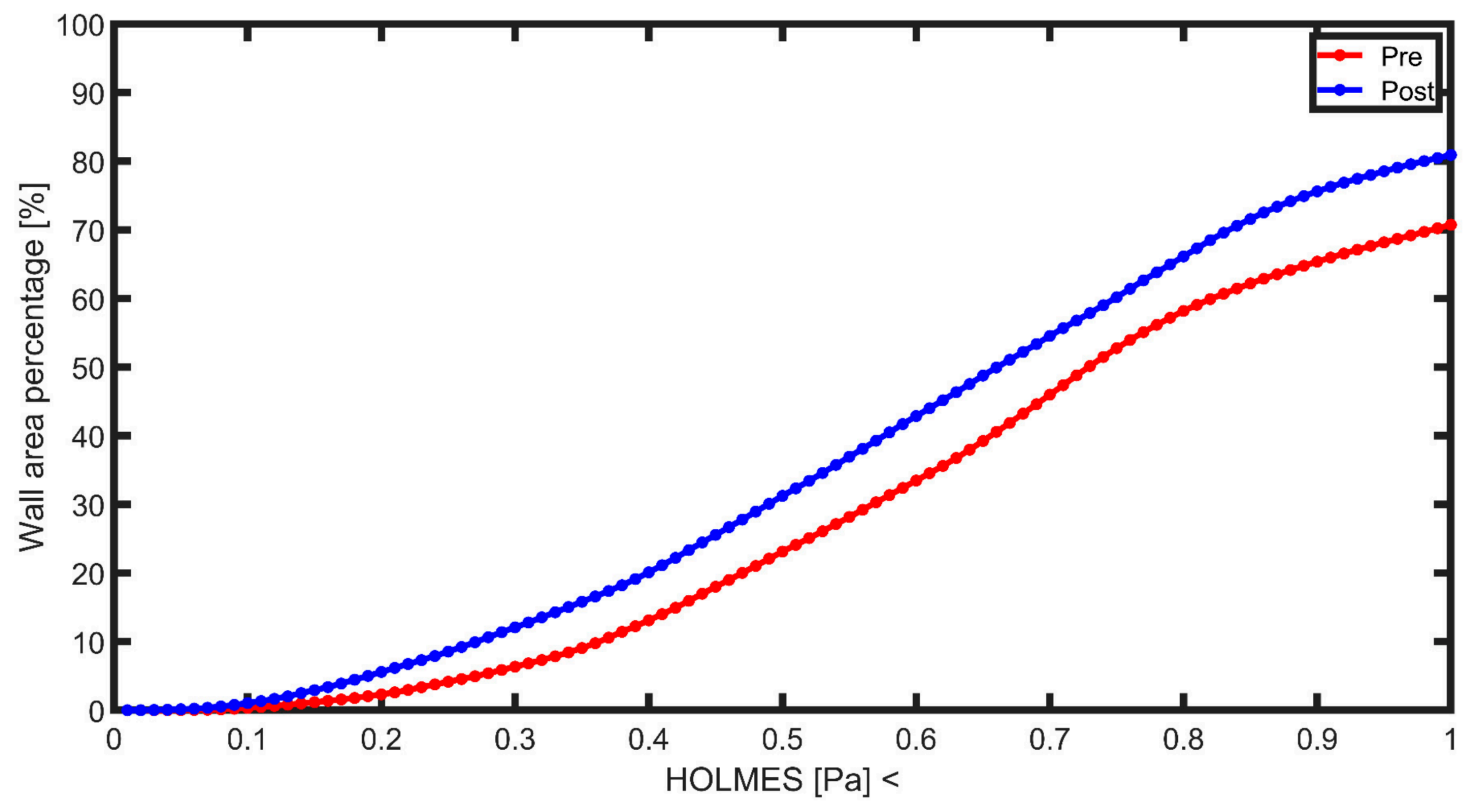
Percentage of the wall area with HOLMES value below H, (H ∈ {0, 0.01, 0.02, …, 1}) for before and after intervention instances.

Low HOLMES values, which have a direct relation with low TAWSS and high OSI in Figure 4e,f, as previously described in Figure 4a–d, are replicated clearly. A noteworthy effect of the intervention has been the increase in areas with HOLMES below 0.3 Pa, which can be seen in the distal region of the stent. Furthermore, this change can also be noted for values other than 0.3 Pa, and this can also be seen in Figure 5.

### 3.4. Swirling Strength Indices

**Figure 6:**
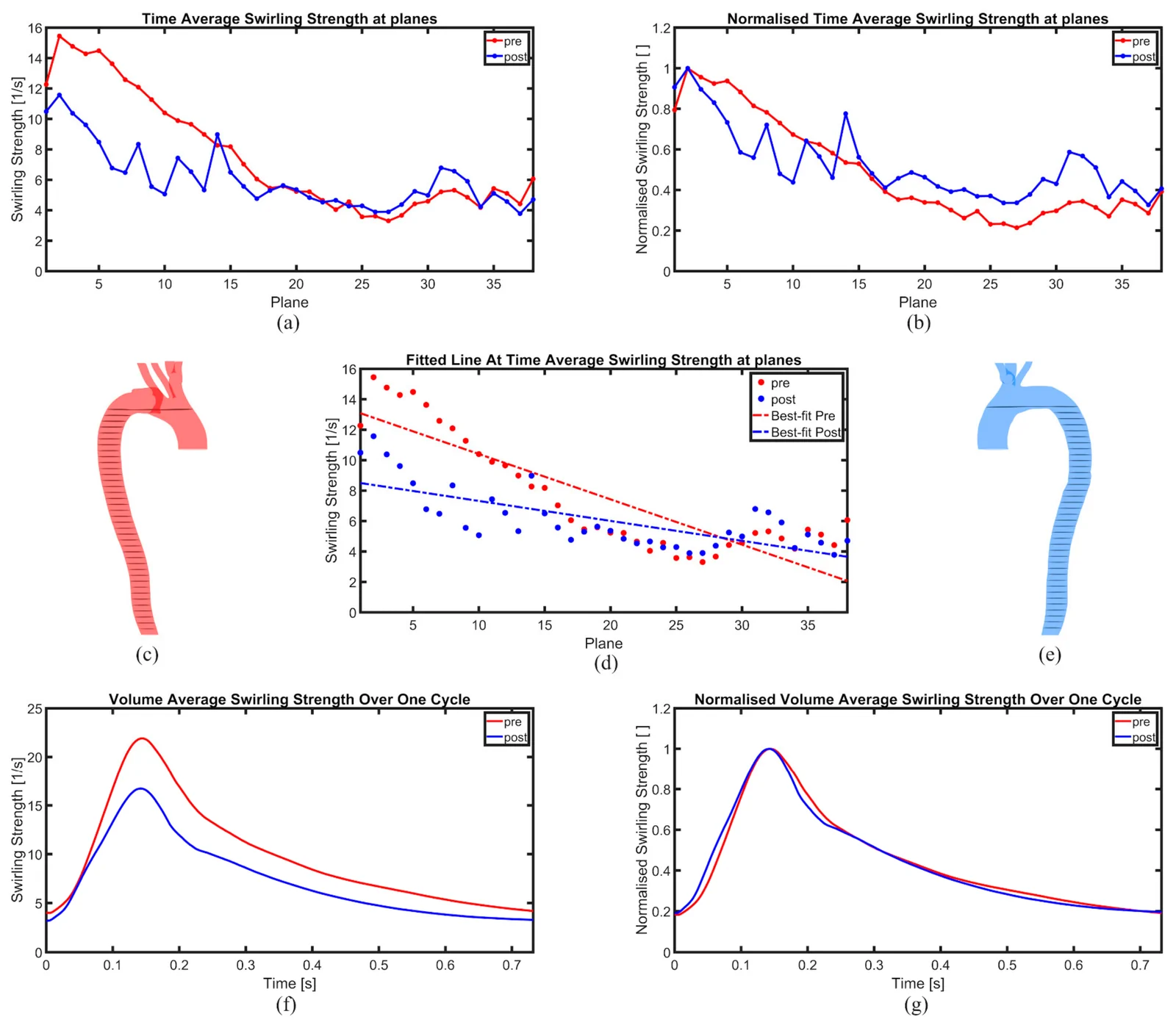
Swirling strength indices and plane locations. (**a**) TASS in both instances, across the planes, (**b**) NTASS in both instances, across the planes, (**c**,**e**) plane locations for pre and post cases, (**d**) TASS values data points and fitted lines for both cases, (**f**) VASS in the whole of the aorta, and (**g**) NVASS in the whole of the aorta.

Figure 6a,b displays TASS and NTASS values across the defined cross-sectional planes for pre- and post-intervention cases. Both metrics decrease progressively toward the distal end of the aorta. Up to the fourteenth plane, which corresponds to the distal end of the stent, TASS significantly decreases post-intervention. Beyond that point, minor differences exist between cases without a clear pattern. In contrast, NTASS shows a distinct increase distal to the fourteenth plane after intervention relative to pre-intervention.

Figure 6d depicts data points and fitted trend lines of TASS for both cases. The post-intervention fit exhibits a less steep slope, indicating reduced variation in SS along the aorta after treatment. This interpretation is supported by the standard deviation values (pre-intervention SD = 1.8610; post-intervention SD = 1.3835). VASS, shown in Figure 6f, decreases following intervention over one cardiac cycle. Although the absolute VASS values change markedly with stenting, the NVASS maintains a similar distribution pattern pre- and post-intervention.

## 4. Discussion

BTAI is a highly lethal form of trauma, with a fatality rate of 80–85% before hospitalization [1,2], most commonly occurring in road traffic accidents [14]. The numerous clinical efforts to develop fast, efficient, optimized, and minimally invasive treatments must be carefully considered [34,35,36]. However, there is a lack of literature exploring less invasive methods and the reduction of side effects using numerical simulations and their clinical applications for BTAI cases [18,30].

In this study, a numerical method was applied to simulate a successful TEVAR case using patient-specific geometry and minimal clinical information. This is the first attempt to investigate hemodynamic parameters resulting from coverage of the LSA due to treatment.

Velocity distribution, as an essential factor for aortic hemodynamics, reaches the maximum in branches and falls to a minimum in the pseudoaneurysm at mid-systole and peak-systole [37]. Flow non-uniformity in the aorta, especially in the arch (i.e., proximal to distal of pseudoaneurysm), is mitigated after pseudoaneurysm and local narrowing removal, which was reported by Duronio et al. [38]. Consequently, velocity decreased in the arch immediately before and after the pseudoaneurysm [39]. During diastole, the highest velocities previously recorded before stenting diminished due to aortic stiffening [40,41]. In addition, Zhu et al. [42] concluded that covered LSA by coil causes increasing perfusion to DA, which is the primary goal of treatment, and cerebral perfusion that was demonstrated in this study.

Beyond altering perfusion fields, intentional LSA coverage is a pivotal endovascular strategy requiring careful clinical trade-offs. Our findings indicate that while LSA coverage effectively manages BTAI pathology by stabilizing downstream SS, it induces systemic and localized pressure increases. In contemporary TEVAR, these alterations underscore the critical balance between securing an adequate proximal landing zone and managing altered flow profiles. Although a single-case rigid model cannot predict absolute clinical event rates, these localized variations highlight why meticulous pre-procedural planning, virtual graft assessment, and a low threshold for prophylactic LSA revascularization are essential.

Investigating pressure values and gradients along the aorta aids in predicting complications, notably hypertension, before and after TEVAR. In this study, during systole, maximum and minimum pressures occurred in the proximal arch and branches, respectively, while during diastole, these extremes occurred in the DA and LCC artery. Notably, after intervention, pressure increased throughout the aorta, consistent with maintained blood volume and increased flow velocity, as supported by Qiao et al. [43]. After stenting, pressure changes were less drastic, particularly in the pseudoaneurysm region, and pressure gradients along the aorta improved [9].

WSS indices, though not directly measurable in vivo, are reliable predictors of atherosclerosis and thrombosis [25]. TAWSS correlated with flow velocity magnitude; after stenting, TAWSS decreased in the pseudoaneurysm and aortic arch in parallel with velocity reductions, while low TAWSS values increased at the entry to the removed pseudoaneurysm. These findings align with those of Buradi et al. [44] and Oliveira et al. [45]. Such reductions lower rupture risk in BTAI and other cardiovascular diseases, as demonstrated in other studies [30,46].

Asymmetry and curvature of the ascending aorta caused differences in TAWSS between the posterior and interior walls [45]. Improved flow uniformity after TEVAR notably adjusted oscillatory shear index (OSI) from proximal to distal pseudoaneurysm regions, where OSI peaked pre-intervention. After treatment, high OSI regions emerged in the DA and initial arch segment, consistent with flow helicity findings by Takehara et al. [47]. HOLMES, a key parameter combining OSI and TAWSS effects, indicates thrombosis risk where values are low [24]. Clinically, identifying these low HOLMES regions provides surgeons with specific target areas to monitor for thrombus formation during post-operative CT follow-ups. Post-intervention, low HOLMES regions, corresponding to high-risk thrombosis sites in the pseudoaneurysm and distal stent, were identified, as reported by Abdoli et al. [48].

SS, together with streamline patterns, effectively quantifies vorticity numerically [10]. TASS and NTASS, accounting for the entire cardiac cycle, showed a descending pattern along the aorta, reflecting distal flow uniformity in both cases [49]. Post-intervention, TASS drastically decreased up to the distal stent edge, reflecting loss of flow helicity due to reduced cross-sectional area. However, downstream stent changes were inconclusive. After normalizing, NTASS increased distally post-intervention, suggesting mitigation of flow irregularities caused by stent grafts and improved perfusion in this successful TEVAR case, supported by other studies [50,51].

In unsuccessful treatments, failure to reduce SS in the stented region and proximal stent can increase WSS, raising rupture risk and atherosclerosis vulnerability [52]. Conversely, excessive SS at the proximal stent end can elevate WSS and rupture risk [30,53]; thus, treatment should avoid both extremes. Marked differences in SS and derivatives (TASS and NTASS) at these sites cause flow and pressure discontinuities, but the low TASS variation observed here indicates relative hemodynamic improvement and clinical success [49]. VASS decreased post-intervention throughout the cardiac cycle, while normalized values (NVASS) remained consistent, following patterns reported by Dadras et al. [30] and Wang et al. [49]. Future studies comparing SS in healthy cases before BTAI may help establish optimal post-surgical SS targets along the aorta.

While this framework successfully captures patient-specific hemodynamic trends, several limitations must be acknowledged. First, due to the single-case design of this study, statistical testing could not be performed to generalize these findings. Consequently, our future work must focus on applying this computational framework to a larger cohort of patients with available and complete datasets to enable rigorous statistical validation. Finally, long-term follow-up beyond 12 months was not available in the present study, limiting assessment of the long-term durability and late complications of TEVAR.

## 5. Conclusions

To the best of our knowledge, this study is the first to perform a numerical simulation investigating the effects of LSA coverage by a stent graft on hemodynamic parameters using pre- and post-procedural images of TEVAR for BTAI cases. While a balanced interpretation requires acknowledging the primary limitations of this framework (including its single-case design, the application of idealized boundary conditions, and the rigid-wall assumption), the findings demonstrate clear baseline trends. The study demonstrates that the damaged segment of the aortic wall (which manifests as a pseudoaneurysm in this specific case) poses a significant threat not only due to the aortic geometry at its location but also because it indirectly causes non-uniform flow patterns distal to its narrowing region. TEVAR effectively mitigates these adverse flow characteristics. Furthermore, after TEVAR, complete exclusion of this damaged segment of the aortic wall and LSA coverage by the stent graft improve the overall pressure distribution but lead to increased pressure throughout the aorta, which may necessitate secondary interventions. The expansion of regions with low HOLMES values post-stenting signals an elevated thrombus risk. Normalized TASS analysis indicates that successful TEVAR modulates SS in the stented region and downstream, stabilizing flow by reducing SS variability. Ultimately, the true value of mapping these localized hemodynamic trends extends beyond abstract computational metrics; it establishes a predictive foundation to support future patient-specific TEVAR planning, enabling clinicians to virtually assess graft configurations and anticipate localized complications. However, translating this framework into routine clinical decision-making strictly necessitates rigorous validation across larger patient cohorts in future clinical studies.

## Figures and Tables

**Table 1:** Calibrated Windkessel parameters for each boundary.

	DA	BT1	BT2	LCC	LSA
R (mmHg s/mL)	0.7757	12.6826	12.8369	20.6312	13.9840
C (ml/mmHg)	2.422	0.501	0.505	0.262	1.062
r (mmHg s/mL)	0.116	0.192	0.189	0.21	0.307

**Table 2:** Carreau-Yasuda viscosity model parameters for non-Newtonian blood model.

$\mu_{0}$ (Pa s)	$\mu_{\infty }$ (Pa s)	$\lambda_{CY}$ (s)	a	m
0.0220	0.0022	0.1100	0.6440	0.3920

**Table 3:** Relative flow distribution for before and after intervention instances for one cycle.

	DA (%)	BT = BT1 + BT2 (%)	LCC (%)	LSA (%)
Prior to intervention	74.04	6.64	9.52	9.57
Post intervention	76.19	10.15	13.54	0
The difference between post-intervention and pre-intervention	2.15	3.51	4.02	−9.57

## Data Availability

The data presented in this study are available upon reasonable request from the corresponding author.

## References

[1] Fox N., Schwartz D., Salazar J.H., Haut E.R., Dahm P., Black J.H., Brakenridge S.C., Como J.J., Hendershot K., King D.R. (2015). Evaluation and Management of Blunt Traumatic Aortic Injury: A Practice Management Guideline from the Eastern Association for the Surgery of Trauma. J. Trauma Acute Care Surg..

[2] Quencer K.B. (2020). Endovascular Interventions in Trauma, an Introduction. Ann. Transl. Med..

[3] Boutin L., Caballero M.-J., Guarrigue D., Hammad E., Rennuit I., Delhaye N., Neuschwander A., Meyer A., Bitot V., Mathais Q. (2022). Blunt Traumatic Aortic Injury Management, a French TraumaBase Analytic Cohort. Eur. J. Vasc. Endovasc. Surg..

[4] Mouawad N.J., Paulisin J., Hofmeister S., Thomas M.B. (2020). Blunt Thoracic Aortic Injury—Concepts and Management. J. Cardiothorac. Surg..

[5] Gaffey A.C., Zhang J., Saka E., Quatromoni J.G., Glaser J., Kim P., Szeto W., Kalapatapu V. (2020). Natural History of Nonoperative Management of Grade II Blunt Thoracic Aortic Injury. Ann. Vasc. Surg..

[6] Al-Thani H., Hakim S., Asim M., Basharat K., El-Menyar A. (2022). Patterns, Management Options and Outcome of Blunt Thoracic Aortic Injuries: A 20-Year Experience from a Tertiary Care Hospital. Eur. J. Trauma Emerg. Surg..

[7] AlQurashi H.E., Alzahrani H.A., Bafaraj M.O., Bosaeed M., Almasabi M., Banhidarah A. (2024). Endovascular Repair in Blunt Thoracic Aortic Injury: A 10-Year Single Center Experience. Cureus.

[8] Sadeghian M., Ebrahimi P., Soltani P., Ghasemi M., Taheri H., Mehrpooya M. (2024). Successful Management of a Delayed Presentation of Traumatic Descending Thoracic Aorta Pseudoaneurysm: A Literature Review Based on a Case Report. Int. J. Emerg. Med..

[9] Rafieianzab D., Abazari M.A., Soltani M., Alimohammadi M. (2021). The Effect of Coarctation Degrees on Wall Shear Stress Indices. Sci. Rep..

[10] Zhang X., Luo M., Fang K., Li J., Peng Y., Zheng L., Shu C. (2020). Analysis of the Formation Mechanism and Occurrence Possibility of Post-Stenotic Dilatation of the Aorta by CFD Approach. Comput. Methods Programs Biomed..

[11] Sunderland K., Zhao F., Jiang J. (2022). Computational Assessment of Hemodynamics Vortices Within the Cerebral Vasculature Using Informational Entropy. Vascular Tissue Engineering: Methods and Protocols.

[12] Gennai S., Leone N., Mezzetto L., Veraldi G.F., Santi D., Spaggiari G., Resch T., Silingardi R. (2023). Systematic Review and Meta-Analysis of Long-Term Reintervention Following Thoracic Endovascular Repair for Blunt Traumatic Aortic Injury. J. Vasc. Surg..

[13] Azizzadeh A., Charlton-Ouw K.M., Chen Z., Rahbar M.H., Estrera A.L., Amer H., Coogan S.M., Safi H.J. (2013). An Outcome Analysis of Endovascular versus Open Repair of Blunt Traumatic Aortic Injuries. J. Vasc. Surg..

[14] Mazzaccaro D., Righini P., Fancoli F., Giannetta M., Modafferi A., Malacrida G., Nano G. (2023). Blunt Thoracic Aortic Injury. J. Clin. Med..

[15] Wei W., Kahn C.J.F., Behr M. (2019). Fluid–Structure Interaction Simulation of Aortic Blood Flow by Ventricular Beating: A Preliminary Model for Blunt Aortic Injuries in Vehicle Crashes. Int. J. Crashworthiness.

[16] Nan J., Rezaei M., Mazhar R., Jaber F., Musharavati F., Zalnezhad E., Chowdhury M.E.H. (2020). Finite Element Analysis of the Mechanism of Traumatic Aortic Rupture (TAR). Comput. Math. Methods Med..

[17] Grave-Capistrán M.A., Prieto-Vázquez A.Y., Torres-SanMiguel C.R. (2021). Aortic Blunt Trauma Analysis during a Frontal Impact. Appl. Bionics Biomech..

[18] Tong F., Lan F., Chen J., Li D., Li X. (2023). Numerical Study on the Injury Mechanism of Blunt Aortic Rupture of the Occupant in Frontal and Side-Impact. Int. J. Crashworthiness.

[19] Stanger O.H., Pepper J.R., Svensson L.G. (2019). Surgical Management of Aortic Pathology.

[20] Ghanem M., Meyer F., Tautenhahn J., Udelnow A., Halloul Z. (2022). Covering/Overstenting of the Left Subclavian Artery (LSA) in Thoracic Endovascular Repair (TEVAR) to Treat Various Thoracic/Thoracoabdominal Aortic Lesions: Is Revascularization of the Left Arm a Must?. Pol. J. Surg..

[21] Alanezi T., Altoijry A., Alsheikh S., Al-mubarak H. (2024). Predicting the Need for Subclavian Artery Revascularization in Thoracic Endovascular Aortic Repair: A Systematic Review and Meta-Analysis. J. Vasc. Surg..

[22] DuBose J., Kundi R., Curi M.A., Eagleton M., Eskandari M.K., Gandhi S., Malgor R.D., Mitchell E.L., Murad M.H., Quiroga E. (2026). The Society for Vascular Surgery Clinical Practice Guideline on the Management of Blunt Thoracic Aortic Injury: Focused Update. J. Vasc. Surg..

[23] Alimohammadi M. (2018). Aortic Dissection: Simulation Tools for Disease Management and Understanding.

[24] Alimohammadi M., Pichardo-Almarza C., Agu O., Díaz-Zuccarini V. (2016). Development of a Patient-Specific Multi-Scale Model to Understand Atherosclerosis and Calcification Locations: Comparison with in Vivo Data in an Aortic Dissection. Front. Physiol..

[25] Abazari M.A., Rafiei D., Soltani M., Alimohammadi M. (2021). The Effect of Beta-Blockers on Hemodynamic Parameters in Patient-Specific Blood Flow Simulations of Type-B Aortic Dissection: A Virtual Study. Sci. Rep..

[26] Eskandari A., Malek S., Jabbari A., Javari K., Rahmati N., Nikbakhtian B., Mohebbi B., Parhizgar S.E., Alimohammadi M. (2025). Enhancing Cardiac Assessments: Accurate and Efficient Prediction of Quantitative Fractional Flow Reserve. Front. Bioeng. Biotechnol..

[27] Rahmati N., Pouraliakbar H., Eskandari A., Javari K., Jabbarinick A., Sadeghipour P., Soltani M., Alimohammadi M. (2025). The Impact of Stenosis Severity on Hemodynamic Parameters in the Iliac Artery: A Fluid–Structure Interaction Study. Bioengineering.

[28] Rahmati N., Eskandari A., Javari K., Jabbari A., Alimohammadi M. (2025). Optimising Haemodynamic Performance: Investigating the Impact of Varying Anastomosis Angles on Blood Flow in Brachiocephalic Fistula. Appl. Eng. Sci..

[29] Riambau V., Böckler D., Brunkwall J., Cao P., Chiesa R., Coppi G., Czerny M., Fraedrich G., Haulon S., Jacobs M.J. (2017). Editor’s Choice—Management of Descending Thoracic Aorta Diseases: Clinical Practice Guidelines of the European Society for Vascular Surgery (ESVS). Eur. J. Vasc. Endovasc. Surg. Off. J. Eur. Soc. Vasc. Surg..

[30] Dadras R., Jabbari A., Asl N.K., Soltani M., Rafiee F., Parsaee M., Golchin S., Pouraliakbar H., Sadeghipour P., Alimohammadi M. (2023). In-Silico Investigations of Haemodynamic Parameters for a Blunt Thoracic Aortic Injury Case. Sci. Rep..

[31] Gijsen F.J.H., van de Vosse F.N., Janssen J.D. (1999). The Influence of the Non-Newtonian Properties of Blood on the Flow in Large Arteries: Steady Flow in a Carotid Bifurcation Model. J. Biomech..

[32] Adrian R.J., Christensen K.T., Liu Z.-C. (2000). Analysis and Interpretation of Instantaneous Turbulent Velocity Fields. Exp. Fluids.

[33] Xiong Q.-Q., Chen Z., Li S.-W., Wang Y.-D., Xu J.-H. (2018). Micro-PIV Measurement and CFD Simulation of Flow Field and Swirling Strength during Droplet Formation Process in a Coaxial Microchannel. Chem. Eng. Sci..

[34] Dinh K., Limmer A., Ngai C., Cho T., Young N., Hsu J. (2021). Blunt Thoracic Aorta Injuries, an Australian Single Centre’s Perspective. ANZ J. Surg..

[35] Pu X., Huang X., Huang L. (2020). Emergency Percutaneous Thoracic Endovascular Aortic Repair for Patients with Traumatic Thoracic Aortic Blunt Injury: A Single Center Experience. Chin. J. Traumatol..

[36] Brown C.V.R., de Moya M., Brasel K.J., Hartwell J.L., Inaba K., Ley E.J., Moore E.E., Peck K.A., Rizzo A.G., Rosen N.G. (2023). Blunt Thoracic Aortic Injury: A Western Trauma Association Critical Decisions Algorithm. J. Trauma Acute Care Surg..

[37] Petuchova A., Maknickas A. (2022). Computational Analysis of Aortic Haemodynamics in the Presence of Ascending Aortic Aneurysm. Technol. Heal. Care.

[38] Duronio F., Di Mascio A. (2023). Blood Flow Simulation of Aneurysmatic and Sane Thoracic Aorta Using OpenFOAM CFD Software. Fluids.

[39] Polanczyk A., Piechota-Polanczyk A., Domenig C., Nanobachvili J., Huk I., Neumayer C. (2018). Computational Fluid Dynamic Accuracy in Mimicking Changes in Blood Hemodynamics in Patients with Acute Type IIIb Aortic Dissection Treated with TEVAR. Appl. Sci..

[40] Büsen M., Arenz C., Neidlin M., Liao S., Schmitz-Rode T., Steinseifer U., Sonntag S.J. (2017). Development of an In Vitro PIV Setup for Preliminary Investigation of the Effects of Aortic Compliance on Flow Patterns and Hemodynamics. Cardiovasc. Eng. Technol..

[41] Schellinger I.N., Naumann J., Hoffmann A., Barnard S.J., Düsing S., Wagenhäuser M.U., Haunschild J., Scheinert D., Hasenfuß G., Etz C.D. (2023). Abdominal Aortic Endograft Implantation Immediately Induces Vascular Stiffness Gradients That May Promote Adverse Aortic Neck Dilatation: Results of A Porcine Ex Vivo Study. J. Endovasc. Ther..

[42] Zhu Y., Li F., Zhang H., Song H., Ma X., Cao L., Zhang W., Guo W. (2022). Hemodynamic Numerical Simulation of Aortic Arch Modular Inner Branched Stent-Graft in Eight Early Patients from the First-in-Human Case Series. Front. Cardiovasc. Med..

[43] Qiao Y., Fan J., Ding Y., Zhu T., Luo K. (2019). A Primary Computational Fluid Dynamics Study of Pre-and Post-Tevar with Intentional Left Subclavian Artery Coverage in a Type b Aortic Dissection. J. Biomech. Eng..

[44] Buradi A., Mahalingam A. (2020). Numerical Analysis of Wall Shear Stress Parameters of Newtonian Pulsatile Blood Flow Through Coronary Artery and Correlation to Atherosclerosis. Advances in Mechanical Engineering.

[45] Oliveira D.C., Laranjo S., Tiago J., Pinto F.F., Sequeira A. (2020). NUMERICAL SIMULATION of DILATION PATTERNS of the ASCENDING AORTA in AORTOPATHIES. J. Mech. Med. Biol..

[46] Martinelli O., Faccenna F., Malaj A., Jabbour J., Venosi S., Gattuso R., Gossetti B., Irace L. (2018). Hypertension, Acute Stent Thrombosis, and Paraplegia 6 Months after Thoracic Endovascular Aortic Repair for Blunt Thoracic Aortic Injury in a 22-Year-Old Patient. Ann. Vasc. Surg..

[47] Takehara Y., Isoda H., Takahashi M., Unno N., Shiiya N., Ushio T., Goshima S., Naganawa S., Alley M., Wakayama T. (2020). Abnormal Flow Dynamics Result in Low Wall Shear Stress and High Oscillatory Shear Index in Abdominal Aortic Dilatation: Initial in Vivo Assessment with 4D-Flow MRI. Magn. Reson. Med. Sci..

[48] Abdoli S., Ham S.W., Wilcox A.G., Fleischman F., Lam L. (2017). Symptomatic Intragraft Thrombus Following Endovascular Repair of Blunt Thoracic Aortic Injury. Ann. Vasc. Surg..

[49] Wang Z., Tian S., Yu L., Ma X., Xing Y., Ma X. (2021). The Role of the Aortic Area in Type A Aortic Dissection. Biomed. Signal Process. Control.

[50] Sun A., Fan Y., Deng X. (2012). Intentionally Induced Swirling Flow May Improve the Hemodynamic Performance of Coronary Bifurcation Stenting. Catheter. Cardiovasc. Interv..

[51] Chen Z., Fan Y., Deng X., Xu Z. (2009). Swirling Flow Can Suppress Flow Disturbances in Endovascular Stents: A Numerical Study. ASAIO J..

[52] Lichtenberg M., Zeller T., Gaines P., Piorkowski M. (2022). BioMimics 3D Vascular Stent System for Femoropopliteal Interventions. Vasa.

[53] Ferdian E., Dubowitz D.J., Mauger C.A., Wang A., Young A.A. (2022). WSSNet: Aortic Wall Shear Stress Estimation Using Deep Learning on 4D Flow MRI. Front. Cardiovasc. Med..

